# The influence of algorithms on political and dating decisions

**DOI:** 10.1371/journal.pone.0249454

**Published:** 2021-04-21

**Authors:** Ujué Agudo, Helena Matute

**Affiliations:** Universidad de Deusto, Bilbao, Spain; West Pomeranian University of Technology, POLAND

## Abstract

Artificial intelligence algorithms are ubiquitous in daily life, and this is motivating the development of some institutional initiatives to ensure trustworthiness in Artificial Intelligence (AI). However, there is not enough research on how these algorithms can influence people’s decisions and attitudes. The present research examines whether algorithms can persuade people, explicitly or covertly, on whom to vote and date, or whether, by contrast, people would reject their influence in an attempt to confirm their personal freedom and independence. In four experiments, we found that persuasion was possible and that different styles of persuasion (e.g., explicit, covert) were more effective depending on the decision context (e.g., political and dating). We conclude that it is important to educate people against trusting and following the advice of algorithms blindly. A discussion on who owns and can use the data that makes these algorithms work efficiently is also necessary.

## Introduction

Every day, new headlines appear in which Artificial Intelligence (AI) has overtaken human capacity in new and different domains, such as recognizing cardiac arrest through a phone call [1], predicting the outcomes of couple therapy better than experts [2], or reducing diagnostic errors in breast cancer patients [3]. This results in recommendation and persuasion algorithms being widely used nowadays, offering people advice on what to read, what to buy, where to eat, or whom to date, and people often assume that these AI judgments are objective, efficient, and reliable [4–6]; a phenomenon often known as *machine bias* [7].

This situation has led to some warnings about how these algorithms and the companies that create them could be manipulating people’s decisions in important ways. In fact, some companies, particularly Facebook and Google, have been blamed for manipulating democratic elections, and more and more voices are calling for stronger regulations on AI in order to protect democracy [8–10]. In response to this problem, some institutional initiatives are being developed. For example, the European Union has recently released the document Ethics Guidelines for a Trustworthy AI, which aims to promote the development of AI in which people can trust. This is described as AI that favors "human agency and oversight", possesses "technical robustness and safety", guarantees "privacy and data governance", provides "transparency", respects "diversity, non-discrimination, and fairness", promotes "social and environmental well-being", and allows "accountability" [11]. At the same time, however, many scholars and journalists are skeptical of these warnings and initiatives. In particular, the scientific literature on acceptance of algorithmic advice, with some exceptions [12], reports a certain aversion to algorithmic advice in society (see [13], for a review, suggesting that most people tend to prefer the advice of a human expert over that provided by an algorithm).

However, it is not only a question of whether AI could influence people through explicit recommendation and persuasion, but also of whether AI can influence human decisions through more covert persuasion and manipulation techniques. Indeed, some studies show that AI can make use of human heuristics and biases in order to manipulate people’s decisions in a subtle way. A famous example is an experiment on voting behavior during the 2010 congressional election in the U.S., using a sample of 61 million Facebook users [14]. The results showed that Facebook messages influenced political self-expression and voting behavior in millions of people. These results were subsequently replicated during the 2012 U.S. Presidential election [15]. Interestingly, successful messages were not presented as mere algorithmic recommendations, but made use of “social proof” [16], pushing Facebook users to vote by imitation, by showing the pictures of those friends of theirs who said they had already voted. Thus, the presentation format exploited a well-known human heuristic (i.e., the tendency to imitate the behavior of the majority and of friends) instead of using an explicit recommendation of the algorithm.

Heuristics are shortcuts of thought, which are deeply configured in the human mind and often allow us to emit fast responses to the demands of the environment without the need for much thinking, data collection, or time and energy consumption. These default reactions are highly efficient most of the time, but they become biases when they guide decisions in situations where they are not safe or appropriate [17]. Indeed, these biases can be used to manipulate thinking and behavior, sometimes in the interest of third parties. In the example above, the algorithm selects the pictures of people who have already voted to show them to their friends (who are the target subjects of the study) in order to manipulate their behavior. According to the authors, using "social proof" to increase voting behavior resulted in the direct participation in the congressional elections of some 60,000 voters and indirectly of another 280,000. Such numbers can tilt the result of any democratic election.

To the best of our knowledge, several other covert manipulations of preferences have also been promoted by exploiting well-known heuristics and biases. For example, manipulating the order in which different political candidates are presented in the Google search results [18], or increasing the familiarity of some political candidates to induce more credibility [19] are strategies that make use of cognitive biases, and thus reduce critical thinking and alerting mechanisms [17]. In consequence, they have been shown to (covertly) attract more votes to their target candidates. Moreover, these subtle influence strategies can make the algorithm’s influence on behavior go unnoticed, and people may often feel that they have made their decision freely even though they might be voting against their own interest.

Publicly available investigations about the potential of AI to influence people’s decisions are still scarce, particularly as compared to the huge amount of private and not published investigations conducted every day by AI-based Internet companies. Companies with potential conflicts of interest are conducting private behavioral experiments and accessing the data of millions of people without their informed consent, something unthinkable for the academic research community [14, 20–22]. Today, their knowledge of what drives human behavior and how to control it is, in order of magnitude, ahead of academic psychology and other social sciences [23]. Therefore, it is necessary to increase the amount of publicly available scientific studies on the influence of AI on human behavior.

The present research aims to empirically test whether an algorithm can influence people’s preferences, either through explicit or covert persuasion, in different contexts. In four experiments we examined whether (a) AI algorithms can influence people explicitly (Experiment 1) and covertly (Experiment 2) in a political context; (b) they can influence people explicitly and covertly in a dating context (Experiment 3); and (c) the effectiveness of that influence might depend, at least partially, on the context of the decision (political vs. dating; Experiment 4). Due to ethical considerations, the dating and political contexts, the potential candidates, as well as the algorithms used in these experiments, were all fictitious.

## Ethics statement

The procedure of the experiments was revised and approved by the Ethics Review Board of the University of Deusto. Written informed consent was not requested because the research was online and harmless, and participation was anonymous and voluntary. The responses collected during the experiment were sent anonymously to the experimenters upon explicit permission by the participant, which was indicated by clicking on a "Submit" button. No personal information was collected.

## Experiment 1: Political context, explicit persuasion

This experiment tested whether an AI algorithm could manipulate peoples’ voting preferences through explicit recommendations or whether, by contrast, people would refuse to follow the algorithm’s recommendations, in an attempt to show their independence and freedom.

Although voting attitudes can be affected by many different variables such as physical aspect, compatibility of personality and values, political agendas, and so on, for the sake of simplicity we decided to use only the pictures of the potential candidates. The physical aspect of a candidate is a variable that is known to strongly influence voting decisions [24, 25], and at the same time, it can be easily controlled for during the experiment.

### Method

#### Participants and apparatus

We recruited 441 participants (46.7% women, 2.5% unknown; ages 18–71, *M* = 39.3, *SD* = 10.7) through social media. We used Twitter to distribute an invitation to participate in a study about the role of psychological processes on political decisions. The invitation was written in Spanish and contained a link to the experiment, which was also conducted in Spanish. Participation was anonymous and voluntary. The computer program randomly assigned all participants to either one of two groups, explicit (*n* = 219) and naïve (*n* = 222). We did not ask for any personal data other than age and gender, nor did we use any cookies or software to collect data without informed consent from participants.

We were not aware of any previous experiment similar to this one, so we could not perform an a priori power analysis to determine the sample size. Thus, we conducted a *post-hoc* sensitivity analysis. This showed that, with this sample size, we obtained a power of 0.90 to detect a small-sized effect (*η*^*2*^_*p*_ = .009) in the differences between groups.

#### Procedure and design

The first part of the experiment (Phase 0) can be described as a “trustworthiness” phase, and was identical for all participants in all four experiments. The computer program instructed all participants that the aim of this phase was to get a better understanding of their personality, so they were asked to answer some questions “from the most important studies in the field of personality”. The purpose of this phase was to give credibility to our fictitious algorithm so that participants would take the "personalized" recommendations they would receive during the experiment seriously. To this end we made use of a version of Forer’s effect [26] similar to that implemented by Barberia, Tubau, Matute and Rodríguez-Ferreiro [27]. It consisted of some dummy personality questions, at the end of which the participants received a supposedly personalized report that, unbeknownst to them, was actually identical for all participants. As in Forer’s experiment, this report was vaguely worded in order to make participants believe that the algorithm had indeed guessed their personality (most participants rated the algorithm as moderately to highly accurate, *M* = 6.71, *SD* = 1.65 in a 1–9 scale).

The design of the experiment is summarized in Table 1. During Phase 1 of the experiment, and in order to reinforce the presumed reliability of the algorithm, all participants observed 32 photographs of (fictitious) political representatives, presented for 1 second each (50% women). The instructions told them that this visualization was necessary for the system to further analyze their personality and preferences, and in order to find those political candidates who would be most appropriate for them. The participants’ task was simply to observe the photos and they were told that they would be asked some questions later on. During Phase 2, all participants were told that, based on their profile, the algorithm would show them eight new political candidates from another country and that their task was to indicate, after a quick look, to what extent they would vote for them if they lived in that country, using a scale from 1 to 9. The pictures of the fictitious politicians (50% women) were presented one at a time, in full-screen format. Because participants were now requested not only to observe each picture but also to type a judgment for each one, the time of exposure was increased to 2 seconds per picture during this phase.

**Table 1:** Design summary of the experiments.

Experiment	Context	Group	Phase 1	Phase 2
**Experiment 1**	Political	Explicit	F1-F32	T1-T4 (*)
				C1-C4
		Naïve	F1-F32	T1-T4
				C1-C4
**Experiment 2**	Political	Covert	F1-F16	T1-T4
			T1-T4 (x4)	C1-C4
**Experiment 3**	Dating	Explicit	F1-F40	T1-T4 (*)
				C1-C4
		Naïve	F1-F40	T1-T4
				C1-C4
		Covert	F1-F20	T1-T4
			T1-T4 (x5)	C1-C4
**Experiment 4**	Political	Explicit	F1-F40	T1-T4 (*)
				C1-C4
		Covert	F1-F20	T1-T4
			T1-T4 (x5)	C1-C4
	Dating	Explicit	F1-F40	T1-T4 (*)
				C1-C4
		Covert	F1-F20	T1-T4
			T1-T4 (x5)	C1-C4

Note. Pictures could be F = Fillers, T = Targets, or C = Controls; (x4) and (x5) = pre-exposed 4 times or 5 times, respectively

(*) = marked as “+90%”.

In the explicit group, four of these pictures (the target stimuli) were explicitly recommended as most compatible with the participant’s personality and preferences, using a badge with the text “+90% compatibility”. The other four pictures were control candidates and did not show the badge. The mean scores for the target candidates and for the control candidates were our dependent variables. In the naïve group, the participants did not receive any suggestions from the algorithm, that is, neither the target nor the control candidates showed any badges. We expected that, in the explicit group, the target candidates should attract more votes than the control candidates, and that in the naïve group participants should show no preferences between target and control candidates.

The pictures of the political candidates were taken from a public database [28]. The order of presentation of each picture within each phase was randomized for each participant. The pictures were counterbalanced in their role as target or control candidates.

### Results and discussion

Age and gender did not affect the results of this experiment, or any of the following ones (all *p*s > .05), so these two variables were collapsed for all analyses presented below (interested readers can download the raw data from the Open Science Framework).

**Fig 1:**
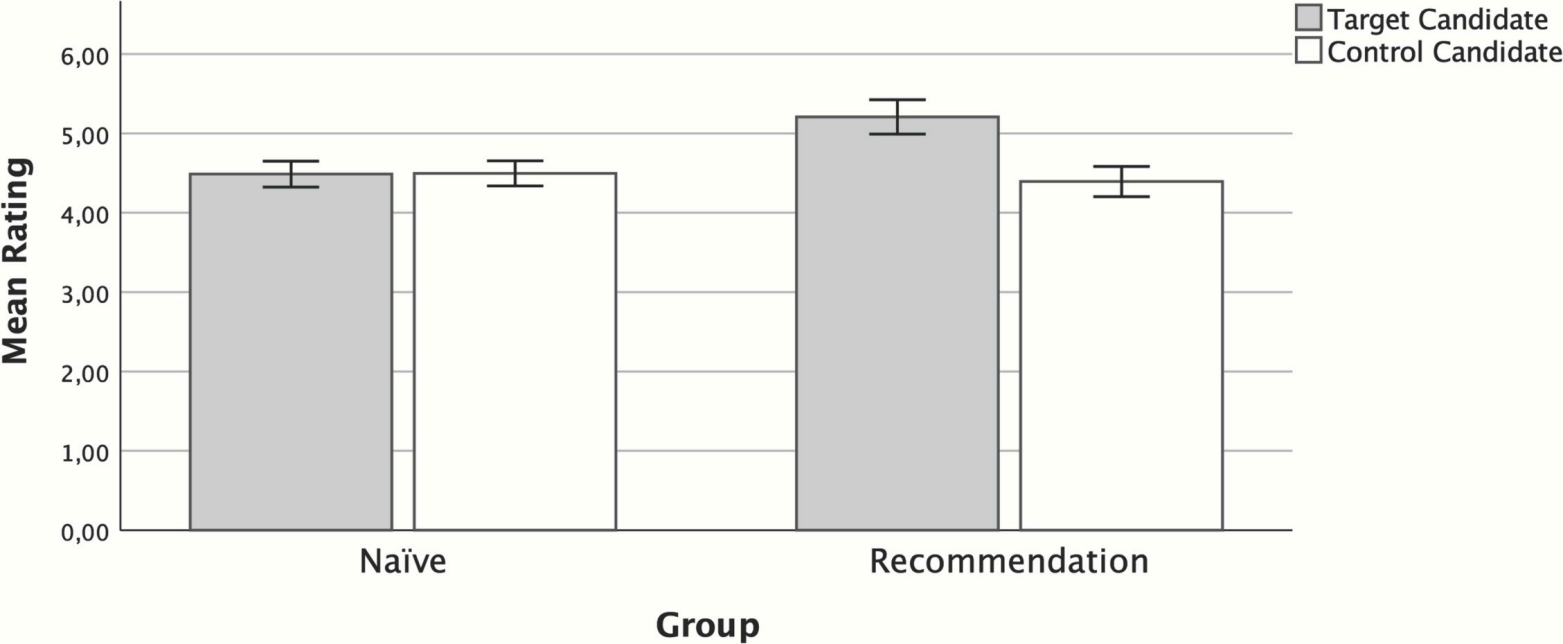
Mean willingness to vote for target and control candidates for each group in Experiment 1. Error bars represent 95% CI.

Fig 1 summarizes the results of this experiment. We conducted a 2 (candidate: target vs. control) x 2 (group: explicit vs. naïve) mixed ANOVA. This ANOVA showed a main effect for group, *F*(1, 439) = 8.15, *p* = .005, *η*^*2*^_*p*_ = .018, a main effect for candidate, *F*(1, 439) = 37.6, *p* < .001, *η*^*2*^_*p*_ = .079, as well as a Group x Candidate interaction, *F*(1, 439) = 42.5, *p* < .001, *η*^*2*^_*p*_ = .088. As expected, *post-hoc* comparisons showed no preference within group naïve for either target or control candidates, *t*(439) = -0.273, *p* = .993, *d* = -0.02. However, and as we expected, participants in group explicit showed a higher willingness to vote for the target candidates than for the control candidates, *t*(439) = 8.913, *p* < .001, *d* = 0.56. (Note that these tests are post-hoc comparisons, so in order to minimize the probability of Type I error, we are comparing the estimated marginal means using the error term and degrees of freedom from the ANOVA [i.e., pooled variances]).

These results showed that, at least in the absence of additional knowledge about the political candidates, the algorithm was able to influence the participants’ voting preferences through a simple explicit recommendation.

## Experiment 2: Political context, covert persuasion

In Experiment 2, we tested whether an AI algorithm could actually influence people’s voting preferences covertly by exploiting their cognitive biases. More specifically, we will try to exploit familiarity bias using the mere-exposure effect. Familiarity is a well-known heuristic that people use to know, in a very short time, and without much data gathering and reasoning, who is reliable and trustable. This heuristic is highly useful, but it becomes a bias when, for instance, people trust the advice of a famous TV actor on whether vaccines are safe, just because familiarity (due to television) makes celebrities appear as trustworthy people by default.

Indeed, many experiments show that mere pre-exposure to a stimulus is a simple way to make it look familiar and acquire attributes related to familiarity [19, 29, 30]. In studies on the effect of mere exposure, participants provide subjective ratings of stimuli that differ in the amount of pre-exposure or familiarity [29]. These experiments show that repeated exposure increases positive responses to the stimuli [31]. The vast existing scientific literature devoted to this field has identified a reliable effect of pre-exposure on subjective ratings, without recognition being a requirement for this effect [30].

Thus, our prediction was that if we could make some candidates look more familiar than others through mere pre-exposure, then pre-exposed candidates would covertly attract more votes than control candidates would.

### Method

#### Participants and apparatus

We recruited 218 participants (48.2% women, 0.7% unknown; ages 30–49, *M* = 35.80, *SD* = 4.46) through Twitter, as in Experiment 1. The public message that we distributed was written in Spanish and contained a link to the experiment, which was also conducted in Spanish. A sensitivity analysis showed that, with this sample size, we obtained a power of 0.90 to detect a small-sized effect (*d* = 0.199).

#### Procedure and design

Experiment 1 had already shown that there were no differences between target and control candidates in the group that was not exposed to recommendation (i.e., group naïve). Thus, in this experiment, we used a within-participants design. That is, we used only one group, exposed to recommendation, but in this case the recommendation was covert rather than explicit. We compared the participants’ ratings of the target candidates vs. the ratings of control candidates.

The design (see Table 1) and the procedure were similar to those of Experiment 1. During Phase 0, the participants completed a dummy personality test and received a presumably individualized personality report. During Phase 1, they observed pictures of (fictitious) political candidates, but this time, four of the pictures (the target candidates) were pre-exposed four times (in 16 trials) in order to produce the familiarity effect or mere-exposure effect. According to Bornstein [29], the size of the exposure effect is greatest when a relatively small number of exposures are used (between one and nine).

During this phase, each participant also observed another 16 pictures as filler stimuli to complete the 32 trials, as in Experiment 1. The 32 trials were presented in pseudorandom order for each participant, following the rule that the repeated stimuli were intermixed with the filler stimuli. To this end, we created four groups of images, with four fillers and four targets each. The images within each of these subgroups were presented randomly and the order of presentation of the groups was also random. The aim was to avoid the repetitions being too obvious and causing rejection or boredom. However, it was not intended that the participants would be unaware of these repetitions, since previous studies had shown that the effect of mere exposure does not require unawareness, and most previous experiments on this subject do not hide repetition [29, 30].

As in the Experiment 1, the candidates were presented for 1 second each. Based on the previous literature on the mere exposure effect, pre-exposure times between 1 and 4 seconds are the ones that produce a greater effect on subsequent preferences according to Montoya, Horton, Vevea, Citkowicz and Lauber [30]. And according to Bornstein [29], strong effects occur with pre-exposure time of even less than 1 second. Therefore, we decided to use the same 1-second pre-exposure time as in the previous experiment.

During Phase 2, the participants observed those four target pictures in addition to four new pictures (i.e., control candidates) for 2 seconds each. To the best of our knowledge, there is no known standard in the literature on the effect of mere exposure in terms of the time that should be used during the evaluation phase. For this reason, we decided to maintain the 2 seconds of the previous experiment. Furthermore, in many of the previous studies on this effect, the time is also limited, with exposures of 2 seconds [31, 32], 1 second [33] or 8 seconds [34], for example. These eight pictures had been counterbalanced in their roles as target or control candidates, and were presented in random order for each participant. During this phase, the participants were asked to indicate their willingness to vote for each of the eight candidates in a 1–9 scale. The means of these ratings for target and control candidates were our dependent variables. The picture dataset was the same as in Experiment 1 [28].

### Results and discussion

A paired samples t-test showed, contrary to our expectations, that in this experiment the participants’ greater willingness to vote for the target candidates (*M* = 4.45, *SD* = 1.45) than the control candidates (*M* = 4.34, *SD* = 1.28) did not reach statistical significance, *t*(217) = 1.58, *p* = .058, *d* = 0.11.

These results suggest that our covert algorithm was not able to influence the participants’ voting preferences. This could be due to many different causes. For instance, it might be that participants’ individual preferences were strong and the algorithm was not good enough to influence them, or that the procedure was not able to capture the participants’ attention during the pre-exposure phase. Thus, perhaps we should test our algorithm with different parameters until we find those that can make it more effective (in case we are not using the best ones). Indeed, because we had to choose the parameters of our manipulation, such as the number of repetition of the target stimuli during Phase 1, through trial and error, we might be able to find better results with a different set of parameters. For instance, in his meta-analysis of the mere exposure effect, Bornstein [29] recommended a number of repetitions between one to nine in order to achieve a moderate to strong effect. In addition, Montoya et al. [30] warn that the duration of the study can negatively affect the results. For all these reasons, we had preferred to use a low number of repetitions (note also that for each extra repetition, we had to include four more trials with filler candidates, thus extending the length of the experiment). Therefore, we had opted for four repetitions in this experiment, but it did not work. Thus, we decided to introduce some changes in the covert procedure in the next experiment. In addition, we also aimed to conduct a replication of the explicit recommendation observed in Experiment 1, while testing its potential generalization to a very different decision context. To this end, we used a dating context in Experiment 3 (instead of a political one).

## Experiment 3: Dating context, explicit and covert persuasion

In order to test the potential generalization of the results observed in Experiment 1 with a political context, in Experiment 3 we used a dating context. Our main prediction was that the algorithm would be able to guide the participants’ choices through its explicit recommendation, as in Experiment 1, but now in the context of a dating website. In addition, and because the covert influence of the algorithm was not effective in Experiment 2, we now tested whether some changes in the covert procedure could make it more effective in influencing the participants’ preferences in the dating decision context, in which the presence of algorithms is common. The majority of the dating websites boast that their sophisticated compatibility algorithms are to the benefit of their users, claiming that they "provide more compatible matches than traditional dating does" [35]. Although there is not enough research that supports that assertion, the number of users on dating websites has largely increased in the last years [35, 36].

### Method

#### Participants and apparatus

We recruited 280 participants (48.2% women, 0.7% unknown; ages 30–49, *M* = 35.80, *SD* = 4.46) through Prolific Academic’s online platform. The invitation targeted users of White/Caucasian ethnicity, and between the ages of 30–49, so that they would match the age and ethnicity of the fictitious dating candidates. The invitation was written in English, and the experiment was conducted in English. The computer program randomly assigned the participants to either one of three groups: explicit (*n* = 94), covert (*n* = 90), and naïve (*n* = 96). A sensitivity analysis showed that, with this sample size, we obtained a power of 0.90 to detect a small-sized effect (*η*^*2*^_*p*_ = 0.021) in the differences between groups.

#### Procedure and design

The experimental design is presented in Table 1. As in the previous experiments, participants filled a dummy personality test during Phase 0, and they were exposed to pictures of (fictitious) candidates during Phase 1. This time, 40 photographs of potential dating candidates (women or men, according to the preference that the participants indicated at the beginning of the experiment) were shown. Each picture was presented for 1 second. In group explicit and group naïve, all 40 pictures were fillers at this stage. In group covert, only 20 pictures were fillers, while the other 20 trials consisted of four target photographs that were pre-exposed five times each, in order to make them look familiar. Because the covert algorithm used in Experiment 2 to induce a familiarity used four repetitions and did not work well, we now used five repetitions for each target picture, following the suggestions of Rhodes, Halberstadt and Brajkovich [37]. They used four repetitions in their mere exposure experiment with averaged composite faces but found no effect on attractiveness, so they suggest using more than four exposures for certain complex stimuli such as faces. The order of presentation of each picture was pseudo-random for each participant, so that target and filler candidates were intermixed (see Experiment 2 for details).

During Phase 2, all participants used a 1–9 scale to indicate their willingness to send a dating web message to four target candidates and four control candidates. The eight candidates used in this phase were the four target candidates, who had already been used in group covert during Phase 1, plus four new control candidates. The eight pictures were counterbalanced in serving as target or control candidates. In group explicit, the target pictures showed a badge with the text "+90% compatibility", as in Experiment 1. There was no manipulation in group naïve in either phase, so we did not expect any differences between target and control pictures in this group, as they had been fully counterbalanced. As in the previous experiments, the eight pictures used in this phase were presented in random order for each participant.

In contrast to Experiments 1 and 2, and in order to emulate a realistic dating context, we did not use time restrictions on the display of the candidates during Phase 2. In addition, the rating scale was marked with the icons of an "x" and a "heart" at both ends, following the style of the famous dating app Tinder.

### Results and discussion

**Fig 2:**
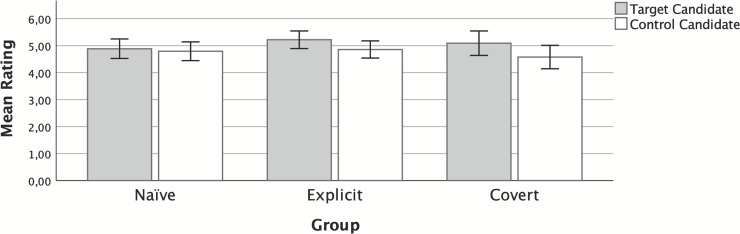
Mean preference for target and control candidates, in the explicit, covert, and no recommendation (naïve) groups in Experiment 3. Error bars represent 95% CI.

The results are depicted in Fig 2. A 2 (candidate: target, control) x 3 (group: explicit, covert, naïve) mixed ANOVA showed a main effect for candidate, *F*(1, 277) = 17.03, *p* < .001, *η*^*2*^_*p*_ = .058, but no main effect for group, *F*(2, 277) = 0.44, *p* = .644, *η*^*2*^_*p*_ = .003, nor a Group x Candidate interaction, *F*(2, 277) = 2.47, *p* = .087, *η*^*2*^_*p*_ = .017.

The main effect of candidate was not relevant to the purpose of this study. It was probably an artifact produced by an "attenuated" interaction in which, as predicted, one group showed the effect and the other groups did not. This main effect is reported for transparency, but is not interpreted.

Thus, for this reason, p*ost-hoc* comparisons were performed to analyze the pairwise comparisons. They showed that in group covert, the participants’ preference was stronger for the target candidates than the control candidates, *t*(277) = 3.72, *p* = .003, *d* = 0.24. However, in group explicit, and contrary to our expectations, the participants did not show a higher willingness to send a dating message to the target than the control candidates, *t*(277) = 2.68, *p* = .083, *d* = 0.24. As expected, there were no differences between the target and the control candidates’ scores in group naïve, *t*(277) = 0.70, *p* = .982, *d* = 0.06.

This experiment shows that manipulating the participants’ preferences toward some candidates covertly in a dating context is relatively easy, making use of human cognitive biases, such as, for instance, increasing the familiarity of these candidates. These results contrast with those of our Experiments 1 and 2. On the one hand, in Experiment 1, we observed that the explicit algorithm influenced people’s willingness to vote for the target political candidates; this result did not replicate in the present experiment using a dating context. On the other hand, the covert algorithm did not work in Experiment 2, but was effective in the current experiment within a dating context.

These results could be due to several factors. On the one hand, they could suggest that the modifications that we implemented in order to improve the covert algorithm with respect to Experiment 2, had been correct, and therefore the covert algorithm is now an effective one. Thus, we could suspect that it could also influence political decisions covertly. Note, however, that many different parameters have been modified between the procedure used in Experiment 3 and the previous experiments, 1 and 2. These are: the language of the study (English vs. Spanish), the recruitment channel of the sample (Prolific Academic vs. Twitter), the number of repetitions in the familiarity bias manipulation (five vs. four), the time restrictions in Phase 2 (no limit vs. 2 seconds), the icons in the scale (present vs. absent), and of course, the different context in which the experiments were framed (dating vs. politics). Any or several of these modifications could have been responsible for the differential results observed. Some of these modifications are procedural parameters that always need to be adjusted in a new procedure, often through trial and error, but others are highly interesting topics that call for further research. Indeed, the differential results that we observed might imply that political decisions can be affected by explicit recommendations, whereas dating decisions might require more subtle manipulations. Thus, in the next experiment, we will test the hypothesis that different decision contexts (e.g., politics vs. dating) might be sensitive to different styles of manipulation (explicit vs. covert).

## Experiment 4. Political and dating contexts, explicit and covert persuasion

Given the results of the previous experiments, we predicted that the way the algorithm would be able to influence the participants’ preferences could depend on context. In principle, algorithmic recommendations could be more effective if they are covert in the dating context, and explicit in the political context. Thus, in this experiment we should be able to replicate the results of Experiment 1 using the explicit manipulation in the political context and the results of Experiment 3 using the covert manipulation in the dating context.

We also considered the possibility that, perhaps, some of the procedural modifications that we included might have also affected the results. For instance, the covert algorithm might have been more effective in Experiment 3 due to the higher number of repetitions of the target candidates and the other modifications that we described. In addition, the explicit algorithm might have been more effective in Experiment 1 due to the time restrictions that we had used in that experiment. Therefore, in this experiment we maintained the parameters that produced the effective explicit manipulation of Experiment 1 and the effective covert manipulation of Experiment 3.

### Method

#### Participants and apparatus

We recruited 400 participants through the Prolific Academic’s online platform (57.5% women, 0.8% unknown). As in Experiment 3, we requested the platform that they forward our invitation to users of White/Caucasian ethnicity and ages 30–45 (*M* = 35.80, *SD* = 4.30), so that they would match the ethnicity and age of the fictitious candidates. The invitation was written in English, and the experiment was conducted in English. The computer program randomly assigned all participants to either one of four groups: political-explicit (*n* = 100), political-covert (*n* = 102); dating-explicit (*n* = 98), dating-covert (*n* = 100). A sensitivity analysis showed that, with this sample size, we obtained a power of 0.90 to detect a small-sized effect (*η*^*2*^_*p*_ = 0.019) in the differences between groups.

#### Procedure and design

The design and the procedure were very similar to those of the previous experiments. We designed a 2 (candidate: target, control) x 2 (group: explicit, covert) x 2 (context: political, dating) experiment. This design is shown in Table 1. During Phase 0, participants filled the dummy personality test. Later, during Phase 1, they were exposed to (fictitious) candidates. Forty photographs of possible candidates were shown. In the dating context, all the photographs were of women or men, according to the preference indicated by the participants. In the political context, half of the candidates were women and half were men. As in Experiment 3, in the explicit group all 40 pictures were fillers and in group covert, only 20 pictures were fillers, while the other 20 trials consisted of four target photographs that were pre-exposed five times each. In this experiment, we changed the photographic data set of the previous experiments and used a more recent one [38] in order to give more realism to the dating context. We also adjusted the color of the compatibility badge of the explicit algorithm so that it would not lose visibility compared to the new pictures. By doing so, we also aimed to test that the results of the previous experiments were not excessively dependent on the particular stimuli that we had used.

During Phase 2, in the dating context, participants used a 1–9 scale (with the icons of an "x" and a "heart" at both ends as in Experiment 3) to indicate their willingness to send a dating web message to each of the eight candidates. In the political context, participants indicated their willingness to vote for them. In both contexts, four of these candidates had been pre-exposed during Phase 1 in the covert group. These were the target candidates. The other four were new control candidates. In the explicit group, the target candidates showed the compatibility badge, while control candidates showed no badge. In all cases, the display time for the to-be-rated candidates was limited to 2 seconds, as in Experiment 1. All pictures were presented in random order for each participant and were counterbalanced in their role as target or control candidates.

### Results and discussion

We first conducted a 2 (Candidate: target vs control) x 2 (Context: political vs. dating) x 2 (Group: explicit vs. covert) mixed ANOVA on the participants’ judgments. This ANOVA showed a triple interaction (Candidate x Group x Context). This is summarized in Table 2.

**Table 2:** Analysis of variance on judgments by group, candidate and context in Experiment 4.

	*df*	*F*	*P*	*η*^*2*^_*p*_
Candidate	1	55.56	< .001	.123
Group	1	0.39	.531	.001
Context	1	53.05	< .001	.118
Group * Candidate	1	0.08	.078	.000
Group * Context	1	0.20	.656	.001
Candidate * Context	1	0.09	.765	.000
Candidate * Group * Context	1	5.61	.018	.014
Total	396			

**Fig 3:**
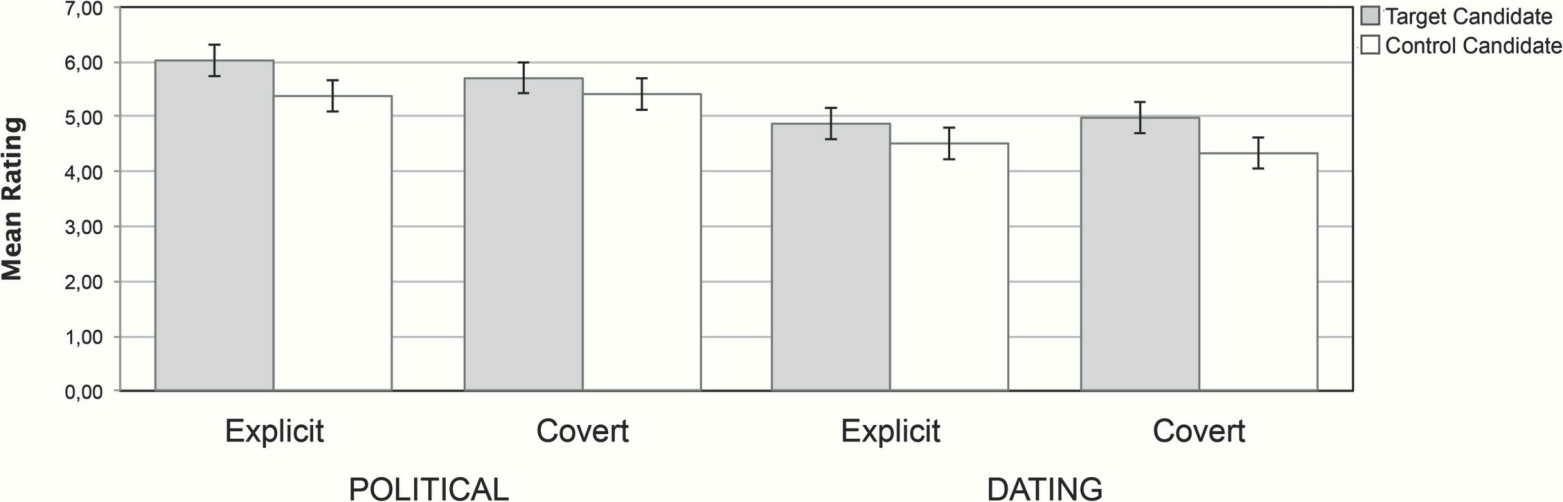
Mean preference for target and control candidates, for each group and context in Experiment 4. Error bars represent 95% CI.

In order to understand the triple interaction, we then conducted the planned comparisons within each decision context. As we expected, the explicit recommendation was effective in the political context, so that participants in the explicit group showed a higher willingness to vote for the target candidates than for the control candidates, *t*(396) = 4.90, *p* < .001, *d* = 0.49. This replicates the results of Experiment 1. Also, and consistent with the results of Experiment 2, no significant differences were observed between target and control candidates when the recommendation was covert in the political context, even though we now were using the 5 repetitions that had been effective in the dating context of Experiment 3, *t*(396) = 2.27, *p* = .310, *d* = 0.26 (Fig 3).

Moreover, on the dating context in this experiment, participants in group covert showed a higher preference for target candidates than for control candidates, *t*(396) = 4.92, *p* < .001, *d* = 0.41, and this difference was not significant in group explicit, *t*(396) = 2.80, *p* = .097, *d* = 0.21. This replicates the results of group covert in Experiment 3. In addition, this suggests that the failure of the explicit algorithm in Experiment 3 to influence the judgments in the dating context was not due to the lack of time restrictions of Experiment 3.

Therefore, it seems that, as expected, the decision context plays a critical role in the effectiveness of explicit and covert algorithm’s persuasion, and confirms the suggestions of previous experiments, with the political decision being more sensitive to explicit recommendations (Experiments 1, 2, and 4) and dating decisions being more strongly affected by the covert manipulation (Experiments 3 and 4). This experiment also shows that the results of the previous experiments were replicated despite our introducing many modifications (different dataset, different badge, and so on).

## General discussion

Some studies have shown that AI can influence people in many different aspects, such as their mood [39], political preferences [18], friendly relationships [40], romantic dates [20], prices paid online [41], and how they spend their time [42]. To the best of our knowledge, however, no one had yet tested the effect of different styles (explicit and covert) of a recommendation algorithm as a function of decision context. Our results indicate that the context can modulate the style of algorithmic persuasion that is more effective in each case. In the particular cases that we tested here, the subtle manipulation that we used was effective on a dating context and the explicit manipulation was effective in a political context. This suggests that very different types of human critical decisions can be manipulated either through explicit or covert algorithm persuasion once the right parameters are known.

In Experiment 1, we observed that the explicit recommendation of our (fictitious) algorithm was able to shape voting preferences in a political context. In Experiment 2, we tried unsuccessfully to influence voting preferences covertly. In Experiment 3, we used a dating context instead of the political one, and implemented several modifications in our covert procedure in an attempt to make it more effective. This experiment showed that covert manipulation of dating preferences could be achieved: Candidates who had been made familiar through pre-exposure were more likely to be highly valued in the context of a dating website. Dating preferences, however, could not be manipulated in Experiment 3 through the explicit recommendation of the algorithm that had been effective in the political context of Experiment 1. These results could be due to the procedural changes that we introduced in Experiment 3, but they could also be due to dating contexts being more vulnerable to covert manipulations and political contexts more vulnerable to explicit manipulations. We tested this hypothesis in Experiment 4.

The results of Experiment 4 replicated and extended those of the previous experiments. They showed that in the political context, our (fictitious) algorithm was able to influence the willingness to vote for some candidates through explicit persuasion (as in Experiment 1), but at the same time our covert algorithm did not influence voting preferences (as in Experiment 2). In addition, Experiment 4 also replicated the results of Experiment 3, showing that in a dating context the covert influence of an algorithm can be powerful in manipulating people’s preferences, while this context is more resistant to explicit persuasion. In addition, the fact that the results of the first three experiments were replicated in Experiment 4 despite the many changes that we introduced (including the picture dataset) suggests that the results are robust.

It might be argued that the experimental context may have seemed unrealistic to the participants, which might have reduced their engagement with the task, because it did not have any real consequences for them. Even so, it should be noted that this potential lack of consequences could not explain our results, as it should affect all conditions equally. However, the participants followed the algorithm’s advice, choosing the target candidates over the control candidates in the experimental conditions and not in the other ones.

It might also be argued that the participants could only rely on the candidates’ photographs, as they were not offered any reference to the political agendas, or the profiles of the candidates in the dating websites. Note, however, that the candidates’ physical appearance was used because it is one of the features that most affects voters [24, 25] and matches on dating websites [20]. Certainly, we could have also manipulated the profiles of the political candidates, particularly their party identification, since this is probably the most influential attribute in voting, as indicated by Bonneau and Cann [43] and Sances [44]. However, we were concerned that in that case our results could be interpreted as a product of this attribute and not of the influence of the algorithm. Thus, we thought it was important to simplify and to reduce the number of variables and potential interactions, given the purpose of our study. Appreciate that our purpose was not to (and our fictitious algorithm could probably not) convince declared leftists or rightists to vote for a candidate of the opposite party, but rather to show that algorithms can influence voting preferences, all other things being equal. Indeed, convinced rightists and lefties may even respond differently to identical stimuli [43, 45–47], so more personalized recommendations should probably be used to influence them.

However, if our fictitious and simplistic algorithm can achieve this level of persuasion on these simple and basic preferences without individualized profiling of the participants, this suggests that a more sophisticated algorithm, such as those with which people interact in their daily life through the Internet, can surely exert a much stronger influence on them. Just as an example, let us imagine the influence that Facebook’s algorithm will be able to reach in its new dating service [48].

As the aim of this study was to empirically test the capacity of algorithms to explicitly and covertly influence peoples’ preferences in important contexts, such as political and dating decisions, the possible causes for the observed differences between the two contexts lay outside the scope of the present research. However, we could speculate that one reason for the significant influence of the explicit algorithm in the political context and the covert algorithm in the dating context might be (at least apparent) the subjective versus objective character of the decisions involved (i.e., more objective in the political context, and more subjective, at least in principle, in the dating context).

Although the results of several previous empirical studies indicate that people generally prefer a human explicit advice to an algorithm explicit advice, the decision context could be playing a critical role. In domains governed by personal taste and preferences (such as, for instance, the dating context), people would prefer a friends’ advice [49, 50], while in more rational environments (such as the political context) the opposite would occur: people would prefer the algorithmic recommendation than the advice of a human [12]. The degree of objectivity attributed to a decision task may perhaps determine in which cases the explicit recommendation of an algorithmic is preferred and in which cases it causes aversion [51]. In those cases in which the algorithm’s explicit advice causes aversion, then the subtle or covert manipulations might nevertheless be used with success, as shown in Experiments 3 and 4.

There are potentially an infinite number of possible variations on the algorithms, their formats, and their parameters. Some variations can produce important differences in the effectiveness of the algorithms, and this raises some questions. Could the covert algorithm influence not only dating decisions but also political decisions if we had used different parameters or if we had aimed to exploit a different cognitive bias instead of the familiarity bias? Since the number of possibilities is so large, we suspect that there must surely exist some combination of parameters that would result in algorithms much more effective than the ones that we presented. Epstein and Robertson [18], for instance, presented political candidates in a particular order in a Google search results page in order to exploit the primacy bias and increase the preference towards one candidate over another. The number of variables that might be changed, and the number of biases that an algorithm could exploit is immense. As shown by our experiments, finding the best possible algorithms is a matter of experimentation. It is important to note, however, that the speed with which human academic scientists can perform new experiments and collect new data is very slow, as compared to the easiness with which many AI companies and their algorithms are already conducting experiments with millions of human beings on a daily basis through the Internet. AI companies can test as many hypotheses as they wish, and with samples as huge as they wish, until they find the most pervasive algorithms. Therefore, their ability to influence decisions both explicitly and covertly is certainly much higher than shown in the present research.

In consequence, we would like to highlight the importance of initiatives such as the Ethics Guidelines for Trustworthy AI, by the European Commission [11], or the DARPA’s explainable AI (XAI) program [52], which aim to increase trustworthiness in AI. However, we believe that a trustworthy AI is only a part of the solution, because increased confidence in AI may also increase the potential hazards as there is not yet enough research to understand how algorithm persuasion affects human decisions [4]. More publicly available experiments are needed to test the effectiveness of different styles of persuasion that algorithms can exert in critical contexts of influence. The present research shows that people are willing to endorse the suggestions of the algorithms and that our own heuristics and biases make us vulnerable to them. Therefore, a human-centric approach [11] should not only aim to establish the critical requirements for AI’s trustworthiness but also to minimize the consequences of that trust on human decisions and freedom. It is of critical importance to educate people against following the advice of algorithms blindly. As a final remark, it is important to highlight the need for a public discussion on who should own the data, which in the end is what is needed make explicit and covert persuasion algorithms work efficiently.
